# Black Hairy Tongue in a 54‐Day‐Old Infant: A Case Report

**DOI:** 10.1002/ccr3.73170

**Published:** 2026-08-04

**Authors:** Masanori Masui, Yumi Sakamoto, Yuki Kunisada, Soichiro Ibaragi

**Affiliations:** ^1^ Department of Oral and Maxillofacial Surgery, Faculty of Medicine, Dentistry and Pharmaceutical Sciences Okayama University Okayama Japan; ^2^ Department of Oral and Maxillofacial Surgery Ako Central Hospital Ako Hyogo Japan

**Keywords:** black hairy tongue, case report, infant, oral hygiene

## Abstract

Black hairy tongue is a benign condition caused by elongation and defective desquamation of the filiform papillae, and it is uncommon in neonates and infants. We report a case in a 54‐day‐old girl who presented with a black lesion on the tongue dorsum. The lesion had first been noted at the 1‐month medical checkup and persisted despite observation. At presentation, a localized blackish‐brown lesion with accentuated filiform papillae was observed on the tongue dorsum and could not be wiped off with gauze. Oral candidiasis was considered in the differential diagnosis, but microbiological examination detected no *Candida* species. A clinical diagnosis of black hairy tongue was made, and the parents were instructed to gently clean the tongue dorsum with gauze and a sponge brush. The lesion resolved within 2 weeks without recurrence. Recognition of this benign entity may help avoid unnecessary treatment in infants.

## Key Clinical Message

Black hairy tongue, although rare in early infancy, should be considered in black lesions on the tongue dorsum. Recognition of its benign nature allows conservative management with oral hygiene and short‐term follow‐up, helping to avoid unnecessary antifungal treatment and invasive investigations.

## 1 Introduction

Black hairy tongue is a benign lesion characterized by a black to brown, hair‐like appearance on the dorsum of the tongue caused by hypertrophy, elongation, and defective desquamation of the filiform papillae. It is usually seen in adults and has been associated with poor oral hygiene, antibiotic or steroid use, xerostomia, smoking, and poor general condition [1].

Among these predisposing factors, medication exposure deserves particular attention when evaluating black hairy tongue. Antibiotics are the drug class most frequently implicated, and black hairy tongue has been reported following treatment with penicillins, cephalosporins, macrolides, metronidazole, and linezolid [2, 3]. Other medications, including drugs associated with xerostomia, antipsychotics, methyldopa, lansoprazole, interferon, and antineoplastic agents, have also been reported in association with this condition [2]. Proposed mechanisms include delayed desquamation of keratinized filiform papillae, alteration of the oral microbial flora, drug‐related discoloration, and oral dryness or reduced mechanical friction [2, 3]. Therefore, a detailed medication history is important when black hairy tongue is suspected.

In contrast, its occurrence in neonates and infants is rare. In infants, the sudden appearance of a dark lesion on the tongue can cause marked anxiety in caregivers. In addition, oral candidiasis and other pigmented lesions may be considered, which can lead to unnecessary medication or invasive examinations. We report a case of black hairy tongue in a 54‐day‐old infant that resolved with conservative oral hygiene measures, and we discuss its diagnostic and clinical significance.

## 2 Case History/Examination

A 54‐day‐old girl was referred to our department with a chief complaint of black discoloration of the tongue. Her family noticed a black lesion on the dorsum of the tongue and consulted a pediatrician at the 1‐month medical checkup. She was followed up by the Department of Pediatrics; however, because no improvement was observed, she was referred to our department at 54 days of age.

There was no remarkable past medical or family history. Her nutritional and general condition were good, and she was being exclusively breastfed. She had received no medications since birth, including antibiotics or corticosteroids. Because she was exclusively breastfed, a maternal medication history was also obtained. The mother reported no use of antibiotics, corticosteroids, or other medications known to be associated with black hairy tongue from delivery until resolution of the lesion.

Intraoral examination revealed a localized blackish‐brown, hairy tongue coating‐like lesion on the dorsum of the tongue that could not be wiped off with gauze (Figure 1). The lesion showed accentuation of the filiform papillae, with no obvious erythema or induration of the surrounding mucosa. Based on these clinical findings, black hairy tongue was suspected.

**FIGURE 1:**
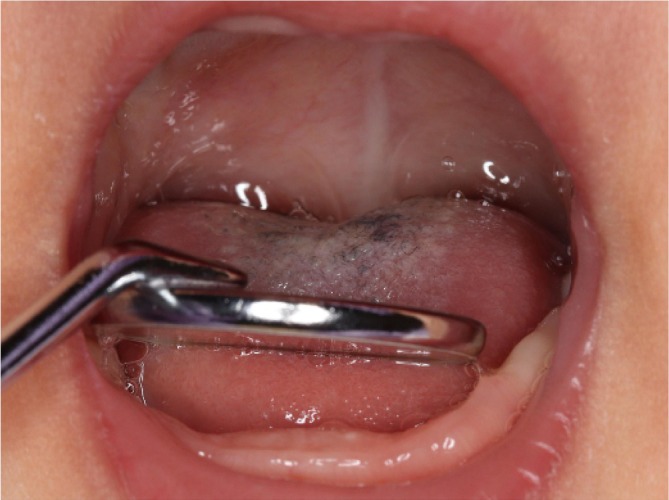
Clinical appearance at the initial visit showing a localized blackish‐brown lesion with a hairy appearance on the dorsum of the tongue.

## 3 Investigations and Treatment

On the day of the 1‐month medical checkup, microbiological examination had been performed in the Department of Pediatrics. Microbiological examination showed epithelial cells 3+, Gram‐positive cocci 2+, and Gram‐positive rods 1+ on smear. Culture revealed *α‐Streptococcus* (++) and *Stomatococcus* species (+), with no *Candida* species detected.

Oral candidiasis was included in the differential diagnosis; however, based on the characteristic clinical findings and the absence of *Candida* species on microbiological examination, a clinical diagnosis of black hairy tongue was made.

The parents were instructed to clean the dorsum of the tongue using gauze and a sponge brush, and the patient was followed up without pharmacological treatment.

## 4 Outcome and Follow‐Up

At the follow‐up visit at 67 days of age, the lesion on the dorsum of the tongue had disappeared, and the mucosal surface was smooth with a normal color (Figure 2). According to the mother, the lesion gradually became thinner and had disappeared before the follow‐up visit. No recurrence has been observed since then.

**FIGURE 2:**
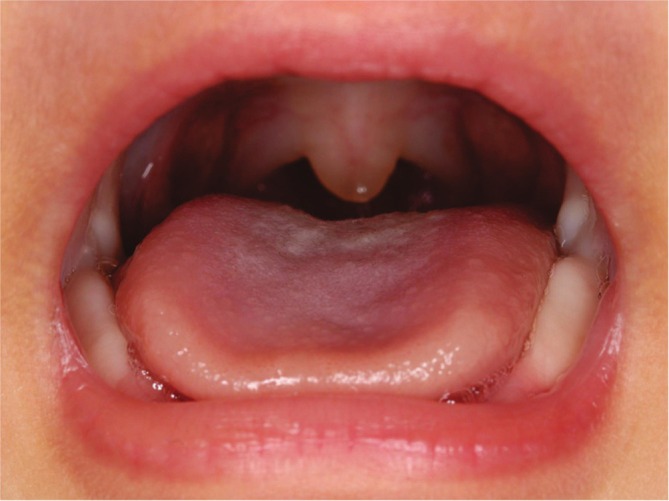
Clinical appearance at follow‐up showing complete resolution of the lesion after oral hygiene instruction.

## 5 Discussion

Black hairy tongue is a benign condition characterized by a black to brown hair‐like appearance caused by hyperkeratosis and defective desquamation of the filiform papillae on the dorsum of the tongue. Poor oral hygiene, smoking, use of antibiotics or steroids, xerostomia, and poor general condition have been reported as associated factors, and the condition is generally observed in adults [1]. In contrast, its occurrence in neonates and infants is rare. To the best of our knowledge, 12 infant cases have been reported previously [4, 5, 6, 7, 8, 9, 10, 11, 12, 13, 14, 15], and together with the present case, 13 cases are summarized in Table 1. The age at onset ranged widely from 2 weeks to 7 months. Reported etiologies include acidification of the oral cavity and tongue surface associated with infant dietary factors such as herbal products or vitamin preparations [4, 11], bacteremia‐induced onset [5], and foreign body‐related onset [9]. However, as in the present case, many reported cases lacked an obvious trigger and were considered to have occurred spontaneously.

**TABLE 1:** Reported cases of black hairy tongue in neonates and infants.

No.	Author (year)	Sex	Age	Duration	Background/risk factor	Suspected etiology	Examination	Treatment	Outcome
1	Poulopoulos et al. (2008) [4]	F	2 months	10 days	Herbal intake	Spontaneous onset	Bacterial examination	Oral hygiene instruction	NR
2	Karadag Oncel et al. (2012) [5]	F	4 months	10 days	Severe growth retardation; immunocompromised status	Bacteremia	Blood culture	Antibiotics (CTRX, VCM)	Resolved in 1 week
3	Körber and Voshege (2012) [6]	F	8 weeks	4 weeks	None	Spontaneous onset	Histopathology	None	Resolved in 3 weeks
4	González Gómez et al. (2012) [7]	F	2 months	20 days	Bronchitis	Spontaneous onset	Bacterial examination	Topical antifungal treatment ineffective; 10% urea topical therapy	Resolved in 1 week
5	Schwartz and Lee (2015) [8]	F	2 weeks	10 days	None	Spontaneous onset	NR	Oral hygiene instruction	Resolved in 3 weeks
6	Oizumi et al. (2016) [9]	F	4 weeks	1 week	None	Foreign‐body related	Bacterial examination	Oral hygiene instruction	Resolved in 6 months
7	Popik et al. (2019) [10]	F	1 month	2 weeks	Gastroesophageal reflux; colic	Spontaneous onset	NR	Topical antifungal treatment ineffective; oral hygiene instruction	Resolved in 2 weeks
8	Owczarek‐Drabińska and Radwan‐Oczko (2020) [11]	F	3 months	3 weeks	Vitamin C supplementation	Spontaneous onset	NR	Oral hygiene instruction	Resolved in 4 months
9	Lopes et al. (2023) [12]	M	24 days	3 days	Anti‐colic herbal extract; previous *Lactobacillus reuteri* supplementation	Dietary factor	None	Discontinuation of anti‐colic herbal extract	Resolved in 2 months
10	Soares et al. (2024) [13]	M	7 months	2 weeks	Homemade onion tea	NR	NR	NR	NR
11	Shah and Hegde (2024) [14]	M	6 months	1 week	None	Spontaneous onset	Tongue scraping	Observation and parental reassurance	Resolved in 4 weeks
12	Dias et al. (2025) [15]	M	5 weeks	1 week	None	Spontaneous onset	NR	Topical antifungal treatment ineffective; oral hygiene instruction	Resolved in 2 weeks
13	Present case (2026)	F	54 days	18 days	None	Spontaneous onset	Bacterial examination	Oral hygiene instruction	Resolved in 2 weeks

Abbreviations: CTRX, ceftriaxone; F, female; M, male; NR, not reported; VCM, vancomycin.

For black‐pigmented lesions on the dorsum of the tongue in infants, oral candidiasis, exogenous pigmentation, and congenital pigmented lesions should be considered in the differential diagnosis. Although black hairy tongue is mainly diagnosed based on typical clinical findings, the extent of the lesion, whether it can be wiped off with gauze, medication history, general condition, and clinical course should all be comprehensively evaluated in infant cases. In the present case, the lesion was localized to the dorsum of the tongue, could not be removed with gauze, and no *Candida* species were detected on microbiological examination; therefore, the diagnosis of black hairy tongue was considered appropriate. In previously reported infant cases, some patients were initially treated with antifungal agents under the impression of oral thrush, but were eventually diagnosed with black hairy tongue after the treatment proved ineffective [7, 10, 15]. Careful differential diagnosis is therefore important to avoid unnecessary drug treatment. Among the previously reported cases, oral microbiological testing at diagnosis was performed in three cases [4, 7, 9], including the present case, it was performed in four cases. Histopathological examination was reported in only one case [6].

With regard to treatment, the basic management of black hairy tongue generally consists of eliminating predisposing factors and improving oral hygiene, with mechanical cleaning of the dorsum of the tongue as the first‐line approach. Most cases improve with these conservative measures, whereas pharmacological treatment or surgical intervention is considered only in persistent or refractory cases [1]. Infant cases have likewise been reported to improve with conservative management. Many cases resolved with observation alone or with oral hygiene instruction centered on gentle cleaning of the tongue using gauze or a brush, whereas antifungal agents were ineffective in some cases. Therefore, in infants, it is appropriate to first explain the benign nature of the lesion to the caregivers and to avoid invasive examinations and unnecessary medication while choosing conservative management. In the present case, the lesion was first noted at the 1‐month medical checkup and showed no improvement during the initial period of observation. However, after referral to our department at 54 days of age and initiation of gentle mechanical cleaning, the lesion resolved within approximately 2 weeks. This clinical course is consistent with the reported turnover time of the oral epithelium, which is approximately 14–24 days [16], and suggests that visible mucosal improvement may require a short period of follow‐up before becoming apparent. In addition, the improvement observed after the start of mechanical cleaning suggests that removal of accumulated keratin and debris may have contributed to epithelial turnover and recovery. Therefore, when black hairy tongue is clinically suspected and no alarming features are present, conservative oral hygiene management combined with short‐term follow‐up may be an appropriate initial strategy, rather than prematurely introducing unnecessary pharmacological treatment or invasive investigations.

In interpreting the microbiological findings in the present case, it is important to consider that the oral environment and oral microbiota in early infancy are still immature. The infant oral microbiota develops under the influence of maternal oral, breast milk, and areolar microbial communities, and before tooth eruption, the available substrates for bacterial adhesion are largely limited to the mucosal epithelium [17, 18]. Recent studies have shown that the oral microbiota develop rapidly during infancy and gradually acquire an adult‐like composition over time [19, 20, 21]. Therefore, the smear and culture findings in a 54‐day‐old infant may reflect the immature oral microbiota of early infancy and should be interpreted cautiously, primarily in the context of differential diagnosis, particularly to exclude oral candidiasis. The core pathology of black hairy tongue is hyperkeratosis and defective desquamation of the filiform papillae, and no specific causative microorganism has been established [1]. The black discoloration has been suggested to result from accumulation of bacteria, fungi, and food debris on elongated filiform papillae, as well as pigment production by chromogenic microorganisms; however, no specific bacterial or fungal species has been identified as the causative agent of black hairy tongue. The *α‐Streptococcus* and *Stomatococcus* spp. detected in the present case were most likely commensal findings reflecting the immature oral microbiota of early infancy, and it would be inappropriate to directly regard them as the primary cause of the lesion. Therefore, in cases of black hairy tongue in infants, microbiological examination should be positioned primarily as a means of differentiating the lesion from oral candidiasis rather than as a method for identifying a causative microorganism.

Taken together, infant black hairy tongue should be diagnosed on the basis of its characteristic clinical findings and clinical course, and managed conservatively.

## 6 Conclusion

We report a case of black hairy tongue in a 54‐day‐old infant. Although rare in early infancy, this condition should be recognized as a benign cause of black discoloration of the tongue dorsum. Awareness of its characteristic clinical features may facilitate appropriate differential diagnosis and help avoid unnecessary antifungal treatment and invasive examinations. Conservative management centered on gentle tongue cleaning appears to be effective.

## Author Contributions

**Soichiro Ibaragi:** writing – review and editing, supervision. **Yuki Kunisada:** writing – review and editing. **Masanori Masui:** conceptualization, investigation, data curation, writing – original draft, visualization. **Yumi Sakamoto:** investigation, data curation, writing – review and editing.

## Funding

The authors have nothing to report.

## Ethics Statement

According to our institutional policy, this case report was exempt from review by the institutional ethics committee because it describes a single patient and contains no identifiable personal information. This report was prepared in accordance with the principles of the Declaration of Helsinki.

## Consent

Written informed consent for publication of this case report and the accompanying images was obtained from the patient's mother.

## Conflicts of Interest

The authors declare no conflicts of interest.

## Data Availability

All data related to the present case are included in this published article.
